# Supplementary material for: Methods of Visualizing the Results of an Artificial-Intelligence-Based Computer-Aided Detection System for Chest Radiographs: Effect on the Diagnostic Performance of Radiologists
Source: Diagnostics (Basel). 2023 Mar 13;13(6):1089. doi: 10.3390/diagnostics13061089 (PMC10046978; doi:10.3390/diagnostics13061089)
Supplement: Supplementary file 1 [file diagnostics-13-01089-s001.zip › diagnostics-2200153-supplementary.pdf]

diagnostics

MDPI

Table S1. Sensitivity, specificity and accuracy of Interpretation of Individual Readers.

Visualization method	Sensitivity	Specificity	
Reader 1			
closed-line method	67.9% (110/162; 60.1–75.0%)	82.8% (72/87; 73.2–90.0%)	73.1% (182/249; 67.1–78.5%)
heat map method	67.3% (109/162; 59.5–74.4%)	82.8% (72/87; 73.2–90.0%)	72.7% (181/249; 66.7–78.1%)
combined method	69.1% (112/162; 61.4–76.1%)	82.8% (72/87; 73.2–90.0%)	73.9% (184/249; 68.0–79.2%)
Reader 2			
closed-line method	74.7% (121/162; 67.3–81.2%)	88.5% (77/87; 79.9–94.3%)	79.5% (198/249; 74.0–84.4%)
heat map method	78.4% (127/162; 70.8–84.0%)	86.2% (75/87; 77.1–92.7%)	81.1% (202/249; 75.7–85.8%)
combined method	78.4% (127/162; 70.8–84.0%)	86.2% (75/87; 77.1–92.7%)	81.1% (202/249; 75.7–85.8%)
Reader 3			
closed-line method	50.0% (81/162; 42.1–57.9%)	90.8% (79/87; 82.7–95.9%)	64.3% (160/249; 58.0–70.2%)
heat map method	54.3% (88/162; 46.3–62.2%)	96.6% (84/87; 90.3–99.3%)	69.1% (172/249; 62.9–74.8%)
combined method	58.6% (95/162; 50.7–66.3%)	94.3% (82/87; 87.1–98.1%)	71.1% (177/249; 65.0–76.6%)
Reader 4			
closed-line method	78.4% (127/162; 70.8–84.0%)	82.8% (72/87; 73.2–90.0%)	79.9% (199/249; 74.4–84.7%)
heat map method	77.8% (126/162; 70.6–83.9%)	85.1% (74/87; 75.8–91.8%)	80.3% (200/249; 74.8–85.1%)
combined method	78.4% (127/162; 70.8–84.0%)	83.9% (73/87; 74.5–90.9%)	80.3% (200/249; 74.8–85.1%)
Reader 5			
closed-line method	66.7% (108/162; 58.8–73.9%)	97.7% (85/87; 91.9–99.7%)	77.5% (193/249; 71.8–82.5%)
heat map method	70.4% (114/162; 62.7–77.3%)	90.8% (79/87; 82.7–95.9%)	77.5% (193/249; 71.8–82.5%)
combined method	69.1% (112/162; 61.4–76.1%)	93.1% (81/87; 85.6–97.4%)	77.5% (193/249; 71.8–82.5%)

Numbers in parentheses indicate numerators/denominators; 95% confidence intervals.

Table S2. Detection Rates of Individual Readers.

Visualization method	All abnormalities	Pulmonary air-space opacity	Pulmonary nodule or mass	Pulmonary interstitial opacity	Pleural effusion	Pneumothorax	Others
Reader 1							
closed-line method	63.3% (112/177; 55.7–70.4%)	69.0% (20/29; 49.2–84.7%)	59.6% (53/89; 48.6–69.8%)	30.8% (4/13; 9.1–61.4%)	90.6% (29/32; 75.0–98.0%)	71.4% (5/7; 29.0–96.3%)	14.3% (1/7; 0.4–57.9%)
heat map method	64.4% (114/177; 56.9–71.4%)	62.1% (18/29; 42.3–79.3%)	61.8% (55/89; 50.9–71.9%)	30.8% (4/13; 9.1–61.4%)	90.6% (29/32; 75.0–98.0%)	100% (7/7; 59.0–100%)	14.3% (1/7; 0.4–57.9%)
combined method	65.5% (116/177; 58.0–72.5%)	55.2% (16/29; 35.7–73.6%)	67.4% (60/89; 56.7–77.0%)	46.2% (6/13; 19.2–74.9%)	84.4% (27/32; 67.2–94.7%)	85.7% (6/7; 42.1–99.6%)	14.3% (1/7; 0.4–57.9%)
Reader 2							
closed-line method	71.2% (126/177; 63.9–77.7%)	75.9% (22/29; 56.5–89.7%)	73.0% (65/89; 62.6–81.9%)	76.9% (10/13; 46.2–95.0%)	71.9% (23/32; 53.3–86.3%)	85.7% (6/7; 42.1–99.6%)	0% (0/7; 0–41.0%)
heat map method	75.7% (134/177; 68.7–81.8%)	65.5% (19/29; 45.7–82.1%)	79.8% (71/89; 69.9–87.6%)	84.6% (11/13; 54.6–98.1%)	81.3% (26/32; 63.6–92.8%)	100% (7/7; 59.0–100%)	0% (0/7; 0–41.0%)
combined method	76.3% (135/177; 69.3–82.3%)	75.9% (22/29; 56.5–89.7%)	78.7% (70/89; 68.7–86.6%)	76.9% (10/13; 46.2–95.0%)	81.3% (26/32; 63.6–92.8%)	100% (7/7; 59.0–100%)	0% (0/7; 0–41.0%)
Reader 3							
closed-line method	45.8% (81/177; 68.3–53.4%)	41.4% (12/29; 23.5–61.1%)	46.1% (41/89; 35.4–57.0%)	38.5% (5/13; 13.9–68.4%)	56.3% (18/32; 37.7–73.6%)	71.4% (5/7; 29.0–96.3%)	0% (0/7; 0–41.0%)
heat map method	49.7% (88/177; 42.1–57.3%)	37.9% (11/29; 20.7–57.7%)	48.3% (43/89; 37.6–59.2%)	53.8% (7/13; 25.1–80.8%)	65.6% (21/32; 46.8–81.4%)	85.7% (6/7; 42.1–99.6%)	0% (0/7; 0–41.0%)
combined method	53.7% (95/177; 46.0–61.2%)	44.8% (13/29; 26.4–64.3%)	57.3 (51/89; 46.4–67.7%)	38.5% (5/13; 13.9–68.4%)	59.4% (19/32; 40.6–76.3%)	100% (7/7; 59.0–100%)	0% (0/7; 0–41.0%)
Reader 4							
closed-line method	73.4% (130/177; 66.3–79.8%)	65.5% (19/29; 45.7–82.1%)	79.8% (71/89; 69.9–87.6%)	69.2% (9/13; 38.6–90.9%)	68.8% (22/32; 50.0–83.9%)	100% (7/7; 59.0–100%)	28.6% (2/7; 3.7–71.0%)
heat map method	72.9% (129/177; 65.7–79.3%)	62.1% (18/29; 42.3–79.3%)	82.0% (73/89; 72.5–89.4%)	61.5% (8/13; 31.6–86.1%)	65.6% (21/32; 46.8–81.4%)	100% (7/7; 59.0–100%)	28.6% (2/7; 3.7–71.0%)
combined method	74.0% (131/177; 66.9–80.3%)	75.9% (22/29; 56.5–89.7%)	79.8% (71/89; 69.9–87.6%)	69.2% (9/13; 38.6–90.9%)	65.6% (21/32; 46.8–81.4%)	100% (7/7; 59.0–100%)	14.3% (1/7; 0.4–57.9%)
Reader 5							
closed-line method	63.8% (113/177; 56.3–70.9%)	55.2% (16/29; 35.7–73.6%)	67.4% (60/89; 56.7–77.0%)	46.2% (6/13; 19.2–74.9%)	78.1% (25/32; 60.0–90.7%)	85.7% (6/7; 42.1–99.6%)	0% (0/7; 0–41.0%)
heat map method	68.9% (122/177; 61.5–75.7%)	58.6% (17/29; 38.9–76.5%)	70.8% (63/89; 60.2–79.9%)	61.5% (8/13; 31.6–86.1%)	84.4% (27/32; 67.2–94.7%)	100% (7/7; 59.0–100%)	0% (0/7; 0–41.0%)
combined method	65.5% (116/177; 58.0–72.5%)	55.2% (16/29; 35.7–73.6%)	68.5% (61/89; 57.8–78.0%)	53.8% (7/13; 25.1–80.8%)	78.1% (25/32; 60.0–90.7%)	100% (7/7; 59.0–100%)	0% (0/7; 0–41.0%)

Abbreviation: AI-CAD, artificial intelligence-based computer-aided detection.
Numbers in parentheses indicate numerators/denominators; 95% confidence intervals.

Table S3. Correlation between Preference, Subject Impression to Improve.

	Overall preference	Subjective impression to improve interpretation accuracy	Sensitivity	Specificity	Accuracy	Detection rate
Reader 1						
closed-line method	5	3	67.9%	82.8%	73.1%	63.3%
heat map method	2	4	67.3%	82.8%	72.7%	64.4%
combined method	4	5	69.1%	82.8%	73.9%	65.5%
Reader 2						
closed-line method	3	4	74.7%	88.5%	79.5%	71.2%
heat map method	4	4	78.4%	86.2%	81.1%	75.7%
combined method	3	4	78.4%	86.2%	81.1%	76.3%
Reader 3						
closed-line method	4	4	50.0%	90.8%	64.3%	45.8%
heat map method	4	4	54.3%	96.6%	69.1%	49.7%
combined method	5	4	58.6%	94.3%	71.1%	53.7%
Reader 4						
closed-line method	4	4	78.4%	82.8%	79.9%	73.4%
heat map method	4	5	77.8%	85.1%	80.3%	72.9%
combined method	5	4	78.4%	83.9%	80.3%	74.0%
Reader 5						
closed-line method	3	5	66.7%	97.7%	77.5%	63.8%
heat map method	4	5	70.4%	90.8%	77.5%	68.9%
combined method	5	5	69.1%	93.1%	77.5%	65.5%
